# Persistence of SARS CoV-2 S1 Protein in CD16+ Monocytes in Post-Acute Sequelae of COVID-19 (PASC) up to 15 Months Post-Infection

**DOI:** 10.3389/fimmu.2021.746021

**Published:** 2022-01-10

**Authors:** Bruce K. Patterson, Edgar B. Francisco, Ram Yogendra, Emily Long, Amruta Pise, Hallison Rodrigues, Eric Hall, Monica Herrera, Purvi Parikh, Jose Guevara-Coto, Timothy J. Triche, Paul Scott, Saboor Hekmati, Dennis Maglinte, Xaiolan Chang, Rodrigo A. Mora-Rodríguez, Javier Mora

**Affiliations:** ^1^ Department of Research and Development, IncellDx Inc, San Carlos, CA, United States; ^2^ Department of Anesthesia, Lawrence General Hospital, Lawrence, MA, United States; ^3^ Department of Molecular Diagnostics, Bio-Rad Laboratories, Hercules, CA, United States; ^4^ Department of Allergy and Immunology, New York University (NYU) Langone Health, New York, NY, United States; ^5^ Lab of Tumor Chemosensitivity, Research Center on Tropical Diseases (CIET)/Research Center on Surgery and Cancer (DC) Lab, Faculty of Microbiology, Universidad de Costa Rica, San Jose, Costa Rica; ^6^ Department of Computer Science and Informatics (ECCI), Universidad de Costa Rica, San Jose, Costa Rica; ^7^ Department of Molecular Biology, Avrok Laboratories, Inc., Azusa, CA, United States; ^8^ Vaccine & Gene Therapy Institute and Oregon National Primate Research Center, Oregon Health & Science University, Portland, OR, United States

**Keywords:** COVID-19, PASC, SARS CoV-2 S1 protein, non-classical monocytes, CCR5, fractalkine

## Abstract

The recent COVID-19 pandemic is a treatment challenge in the acute infection stage but the recognition of chronic COVID-19 symptoms termed post-acute sequelae SARS-CoV-2 infection (PASC) may affect up to 30% of all infected individuals. The underlying mechanism and source of this distinct immunologic condition three months or more after initial infection remains elusive. Here, we investigated the presence of SARS-CoV-2 S1 protein in 46 individuals. We analyzed T-cell, B-cell, and monocytic subsets in both severe COVID-19 patients and in patients with post-acute sequelae of COVID-19 (PASC). The levels of both intermediate (CD14+, CD16+) and non-classical monocyte (CD14Lo, CD16+) were significantly elevated in PASC patients up to 15 months post-acute infection compared to healthy controls (P=0.002 and P=0.01, respectively). A statistically significant number of non-classical monocytes contained SARS-CoV-2 S1 protein in both severe (P=0.004) and PASC patients (P=0.02) out to 15 months post-infection. Non-classical monocytes were sorted from PASC patients using flow cytometric sorting and the SARS-CoV-2 S1 protein was confirmed by mass spectrometry. Cells from 4 out of 11 severe COVID-19 patients and 1 out of 26 PASC patients contained ddPCR+ peripheral blood mononuclear cells, however, only fragmented SARS-CoV-2 RNA was found in PASC patients. No full length sequences were identified, and no sequences that could account for the observed S1 protein were identified in any patient. That non-classical monocytes may be a source of inflammation in PASC warrants further study.

## Introduction

Post-acute sequelae SARS-CoV-2 infection (PASC) is a disabling and sometimes debilitating conditions that occurs in 10%-30% of individuals infected by SARS-CoV-2 and has recently been proposed to cause neurologic symptoms in 30% of those infected (1). The number and extent of symptoms is extremely heterogeneous with some reports suggesting >200 different symptoms (2). The underlying cause of PASC symptoms has remained a mystery though some data has pointed to tissue reservoirs of persistent SARS-CoV-2 as a potential mechanism (3, 4). We recently reported a machine learning approach that identified the unique immunologic signature of individuals with PASC (5). In the same report, we also identified characteristic immune cell subset abnormalities that accompanied the unique cytokine/chemokine profile. The predominant immune cell abnormality was elevations in monocyte subsets (5). Monocyte subpopulations are divided into 3 phenotypic and functionally distinct types. Classical monocytes exhibit the CD14++, CD16- phenotype, intermediate monocytes exhibit a CD14+, CD16+ phenotype, and the non-classical monocytes express CD14lo, CD16+ (6, 7). Further they express very different cell surface markers as previously described (8). In particular, classical monocytes express high levels of the ACE-2 receptor, the putative receptor for SARS-CoV-2 (8). Intermediate and non-classical monocytes express very little ACE-2 receptor. Similarly, classical monocytes express low levels of the chemokine receptors CX3R1 and CCR5. Intermediate monocytes express high levels of CCR5 while non-classical monocytes express high levels of CXC3R1. Here, we report kinetic differences in the proportions of monocyte subsets in severe cases and PASC, as well as the presence of SARS-CoV-2 protein unaccompanied by corresponding viral RNA in CD14lo, CD16+ monocytes in PASC patients up to 15 months post-acute SARS-CoV-2 infection.

## Material/Methods

### Patients

Following informed consent, whole blood was collected in a 10 mL EDTA tube and a 10 mL plasma preparation tube (PPT). A total of 46 individuals were enrolled in the study consisting of 8 healthy controls, 11 severe COVID, 1 asymptomatic, 26 post-COVID individuals. Long Haulers symptoms were previously published (5). Study subjects were stratified according to the following criteria.

Mild:

1. Fever, cough, sore throat, malaise, headache, myalgia, nausea, diarrhea, loss of taste and small
2. No sign of pneumonia on chest imaging (CXR or CT Chest)
3. No shortness of breath or dyspnea

Moderate:

1. Radiological findings of pneumonia fever and respiratory symptoms
2. Saturation of oxygen (SpO2) ≥ 94% on room air at sea level

Severe:

1. Saturation of oxygen (SpO2) < 94% on room air at sea level
2. Arterial partial pressure of oxygen (PaO2)/fraction of inspired oxygen (FiO2) < 300mmHG
3. Lung infiltrate > 50% within 24 to 48 hours
4. HR ≥ 125 bpm
5. Respiratory rate ≥ 30 breaths per minute

Critical:

1. Respiratory failure and requiring mechanical ventilation, ECMO, high-flow nasal cannula oxygen supplementation, noninvasive positive pressure ventilation (BiPAP, CPAP)
2. Septic Shock- Systolic blood pressure < 90mmHg or Diastolic blood pressure < 60 mmHg or requiring vasopressors (levophed, vasopressin, epinephrine
3. Multiple organ dysfunction (cardiac, hepatic, renal, CNS, thrombotic disease)

Post-acute COVID-19 (Long COVID)

1. Extending beyond 3 weeks from the initial onset of first symptoms

Chronic COVID-19:

1. Extending beyond 12 weeks from the initial onset of first symptoms

### High Parameter Immune Profiling/Flow Cytometry

Peripheral blood mononuclear cells were isolated from peripheral blood using Lymphoprep density gradient (STEMCELL Technologies, Vancouver, Canada). Aliquots of 200,000 cells were frozen in media that contained 90% fetal bovine serum (HyClone, Logan, UT) and 10% dimethyl sulfoxide (Sigma-Aldrich, St. Louis, MO) and stored at -70°C for <1 week to preserve viability and run as a single batch to avoid intrarun variability. Cells were blocked with 2% BSA solution for 5 min. at RT. A cocktail containing the following antibodies and reagents was added: Brilliant Stain Buffer (BD Biosciences, San Jose, CA), True-Stain Monocyte Blocker, anti-CD19-PE-Dazzle594, anti-CTLA-4 PE-Cy7, anti-CD3-APC, anti-CD16-Alexa Fluor 700 (all BioLegend, San Diego, CA), and anti-SARS-CoV-2 Spike S1 Subunit-Alexa Fluor 405 (R&D Systems, Minneapolis, MN). The following antibodies were then added: anti-CD8 BUV496, anti-CD4-BUV661, anti-CD45-BUV805, anti-PD-1-BB700 (all BD Biosciences, San Jose, CA), anti-CD56-BV711 (BioLegend, San Diego, CA), and anti-CD14 BV786 (BioLegend, San Diego, CA). Cells were stained for 30 min. at RT, and then washed twice with 2%BSA. Cells were fixed for 1 hour at RT in 1 mL incellMAX (IncellDx, San Carlos, CA), and then incubated with anti-FoxP3-PE antibody (BD Biosciences, San Jose, CA) for 30 min. Cells were washed twice with 2%BSA and then acquired on a 5-laser CytoFLEX LX.

### Digital Droplet PCR

A QIAamp Viral Mini Kit (Qiagen, Germantown, MD) was used to extract nucleic acids from 300 to 400mL of plasma sample according to the manufacturer’s instructions and eluted in 50 mL of AVE buffer (RNase-free water with 0.04% sodium azide). The purified nucleic acids were tested immediately with a Bio-Rad SARS-CoV-2 ddPCR Kit (Bio-Rad, Hercules, CA, USA). The panel was designed for specifically detecting 2019-nCoV (two primer/probe sets). An additional primer/probe set was used to detect the human RNase P gene in control samples and clinical specimens. RNA isolated and purified from the plasma samples (5.5 mL) was added to a master mix comprising 1.1 mL of 2019-nCoV triplex assay, 2.2 mL of reverse transcriptase, 5.5 mL of supermix, 1.1 mL of dithiothreitol, and 6.6 mL of nuclease-free water.

The mixtures were then fractionated into up to 20,000 nanoliter-sized droplets in the form of a water-in-oil emulsion in a QX200 Automated Droplet Generator (Bio-Rad, Hercules, CA). The 96-well real-time-digital droplet polymerase chain reaction (RT-ddPCR) ready plate containing droplets was sealed with foil using a plate sealer and thermocycled to reverse transcribe the RNA, before PCR amplification of cDNA in a C1000 Touch thermocycler (Bio-Rad, Hercules, CA, USA). After PCR, the plate was loaded into a QX200 Droplet Reader (Bio-Rad, Hercules, CA, USA) and the fluorescence intensity of each droplet was measured in two channels (FAM and HEX). The fluorescence data were then analyzed with QuantaSoft 1.7 and QuantaSoft Analysis Pro 1.0 Software (Bio-Rad, Hercules, CA, USA).

### Flow Cytometric Cell Sorting

Cryopreserved PBMCs were quick-thawed, centrifuged, and washed in 2% BSA solution in D-PBS. Cells were blocked for 5 min. in 2% BSA and then incubated at room temperature for 30 min. with Alexa Fluor^®^ 488 Anti-CD45 antibody (IncellDx, 1/100 dilution), 2.5 ug of Alexa Fluor^®^ 647 Anti-CD16 antibody (BD, Cat. # 55710), and 1 ug of PerCP/Cy5.5 Anti-human CD14 antibody (Biolegend, Cat. #325622). Cells were washed twice with 2% BSA/D-PBS, filtered, and kept on ice for the duration of the cell sort. Data was acquired on a Sony SH800, and only CD45+ cells staining positive for both CD14+ and CD16+ were sorted into test tubes with 100 uL 2% BSA solution. Sort purity of control PBMCs was confirmed to be >99% by re-analyzing sorted PBMCs using the same template and gating strategy.

### Single Cell Protein Identification

Patient cells were sorted based on phenotypic markers (as above) and frozen at -80o C. Six patient samples with positive flow cytometry signal and sufficient cell counts were chosen for LCMS confirmation. Frozen cells were lysed with the IP Lysis/Wash Buffer from the kit according to the manufacturer’s protocol. 10 ug of anti-S1 mAb were used to immunoprecipitate the S1 Spike protein from cell lysate of each patient. After overnight incubation with end-over-end rotation at 4oC and then three washes with IP Lysis/Wash Buffer, bound S1 Spike protein was eluted with the elution buffer from the kit.

IP elution fractions were dried in vacuo, resuspended in 20 uL of water, pooled, and purified by Agilent 1290 UPLC Infinity II on a Discovery C8 (3cm x 2.1 mm, 5 µm, Sigma-Aldrich, room temperature) using mobile phase solvents of 0.1% trifluoroacetic acid (TFA) in water or acetonitrile. The gradient is as follows: 5-75% acetonitrile (0.1% TFA) in 4.5 min (0.8 mL/min), with an initial hold at 5% acetonitrile (0.1% TFA) for 0.5 min (0.8 mL/min). The purified protein was dried in vacuo and resuspended in 50 µL of 100 mM HEPES, pH 8.0 (20% Acetonitrile). 1µL of TCEP (100 mM) was added and the samples were incubated at 37°C for 30 min. 1 µL of chloroacetamide (500 mM) was added to the samples and incubated at room temperature for 30 min. 1 µL rAspN (Promega 0.5 µg/µL) and 1 µL of LysC (Pierce, 1 µg/µL) were added and the samples incubated at 37°C for 16 h, prior to LCMS analysis.

### Liquid Chromatography/Mass Spectroscopu (LC-MS) Analysis

Digested recombinant SARS-CoV-2 Spike S1 protein was analyzed by a high accuracy mass spectrometer to generate a list of detectable peptides with retention time and accurate masses. An Agilent 1290 Infinity II high pressure liquid chromatography (HPLC) system and an AdvanceBio Peptide Mapping column (2.1 × 150 mm, 2.7 μm) were used for peptide separation prior to mass analysis. The mobile phase used for peptide separation consists of a solvent A (0.1% formic acid in H_2_O) and a solvent B (0.1% formic acid in 90% CH_3_CN). The gradient was as follows: 0–1 min, 3% B; 1– 30 min, to 40% B; 30–33 min, to 90% B; 33-35 min, 90% B; 37-39 min, 3% B. Eluted peptides were electrosprayed using a Dual JetStream ESI source coupled with the Agilent 6550 iFunnel time-of-flight MS analyzer. Data was acquired using the MS method in 2 GHz (extended dynamic range) mode over a mass/charge range of 50–1700 Daltons and an auto MS/MS method. Acquired data were saved in both centroid and profile mode using Agilent Masshunter Workstation B09 Data acquisition Software. The same analytical method was applied to immunoprecipitated samples from sorted patient cells except no ms/ms was acquired.

### Viral Genome Detection by PCR and Whole Viral Genome Sequencing

#### Ct Determination With TaqPath Assay

Five RNA samples from the sorted populations described above were subjected to the TaqPath COVID-19 Combo Kit Assay (Thermo Fisher Scientific Catalog no. A47814) to assess the cycle of threshold. TaqPath COVID-19 Combo Kit assay was performed according to recommendations of the EUA, using the Applied BioSystems QuantStudio 7 Flex (Thermo Fisher Scientific Catalog no. 4485701).

#### Whole Genome Sequencing of Samples With Ion AmpliSeq

Five RNA samples were subjected to AmpliSeq library preparation using the Ion AmpliSeq Library Kit 2.0 (Thermo Fisher Scientific Catalog no. 4480441) and the Thermo Fisher Scientific Insight panel, which consists of 238 amplicons in a two pool design against SARS-CoV-2 and seven amplicons as human controls. Libraries were prepared following manufacturer’s recommendations. Final libraries were amplified using 5 cycles of amplification and libraries were cleaned up using 0.5X right sided cleanup and 1.2X left sided cleanup using Kapa Pure Beads (Roche Catalog no l7983298001). Final libraries were quantified using Ion Library TaqMan Quantitation Kit (ThermoFisher Catalog no. 4468802). Samples were pooled in an equimolar distribution and loaded on to the Ion Chef Instrument (ThermoFisher Catalog no. 4484177) for Templating onto a 510 chip. The prepared chip was then loaded onto a GeneStudio S5 Prime (ThermoFisher Catalog no. A38196) for sequencing.

### Genome Assembly, Quality Control, and Sequencing Analysis

Sequencing reads were aligned to the SARS-CoV-2 genome (build NC_045512.2) and human transcriptome (build GRCh37) using the Thermo Fisher Scientific TMAP aligner. Default parameters were used except for the –context flag.

Coverage analysis was performed by the coverage Analysis plugin in Thermo Fisher Scientific Torrent Suite software. Reads in the human controls were evaluated for quality control. Per-base coverage, average coverage, and percent genome covered at various depth thresholds were assessed using custom software. Read length distribution versus read quality (MAPQ score) were further evaluated. Variant calling was performed on SARS-CoV-2 using the variantCaller plugin. Callable regions were identified as regions with read depth >= 20 after filtering reads with MAPQ < 10. Variants were filtered for quality by removing mutations with allele frequency (AF) < 0.5 in the callable regions. Lineage determination was made with pangoLEARN v1.2.13 using filtered-in mutations.

## Results

**Figure 1:**
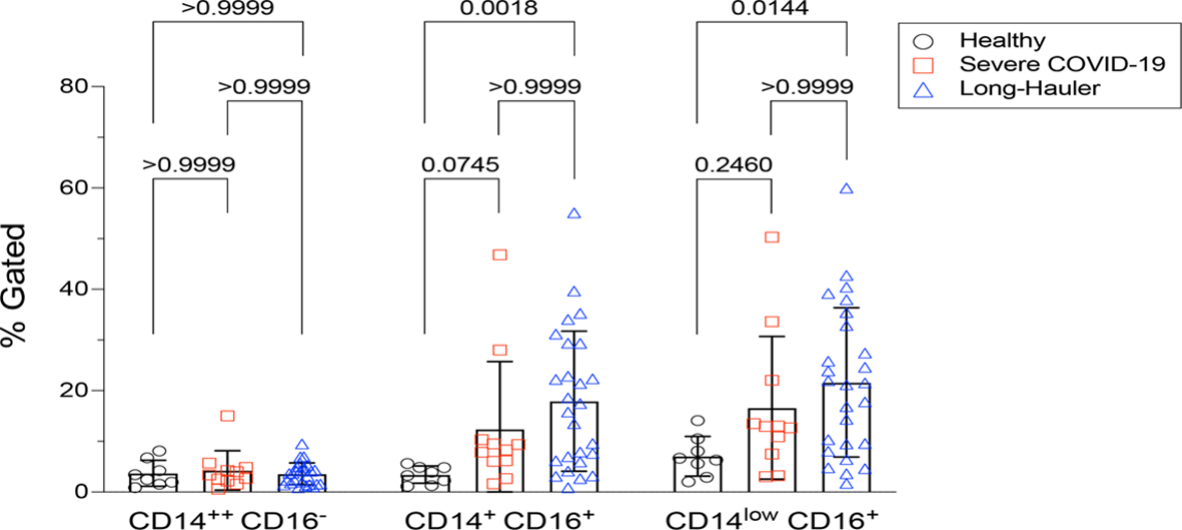
Quantification of classical, intermediate and non-classical monocytes in PASC (LH). Non-classical monocytes were significantly elevated in severe COVID-19 and in PASC. Values for each category were based on the parental gating in **Supplementary Figure 1**. Statistical analysis was performed using the Kluskal-Wallis test. P values <0.05 are considered significant.

Similar to other inflammatory and infectious conditions such as sepsis, lupus erythematosis, and rheumatoid arthritis among others (9), we detected statistically significant increases (P<0.002) of intermediate CD14+, CD16+ monocytes in individuals with PASC compared to healthy controls. In addition, CD14lo, CD16+ non-classical monocytes were also significantly elevated in PASC (P=0.01). Neither intermediate nor non-classical monocytes were elevated in severe COVID-19 (**Figure 1**).

Since the reports by our group and others found that monocyte subsets can be infected by HIV, HCV, Zika virus and Dengue fever virus (10–12), we screened peripheral blood mononuclear cells (PBMCs) from PASC individuals, as well as acute severe COVID-19 as controls, for SARS-CoV-2 RNA (**Table 1**). Using the highly sensitive, quantitative digital droplet PCR (ddPCR), we found that 36% (4 of 11) of severe COVID-19 patients’ PBMCs contained SARS-CoV-2 RNA compared to 4% (1/26) of PASC patients’ PBMCs. The one PASC patient that was RNA positive was 15 months post infection.

**Table 1:** Molecular analysis and duration post-infection of study participants.

COVID-19 Status	Sars-CoV-2 RNA+		Months Post-Infection
	NS	PBMCs	
HC 1	–	–	n/a
HC 2	–	–	n/a
HC 3	–	–	n/a
HC 4	–	–	n/a
HC 5	–	–	n/a
HC 6	–	–	n/a
HC 7	–	–	n/a
HC 8	–	–	n/a
Asymptomatic	+	+	n/a
Severe 1	+	–	n/a
Severe 2	+	+	n/a
Severe 3	+	–	n/a
Severe 4	+	–	n/a
Severe 5	+	–	n/a
Severe 6	+	–	n/a
Severe 7	+	+	n/a
Severe 8	+	–	n/a
Severe 9	+	–	n/a
Severe 10	+	+	n/a
Severe 11	+	+	n/a
LH 1	+	–	13
LH 2	+	–	14
LH 3	+	–	6
LH 4	+	–	11
LH 5	+	+	15
LH 6	+	–	13
LH 7	+	–	12
LH 8	+	–	7
LH 9	+	–	14
LH 10	+	–	13
LH 11	+	–	12
LH 12	+	–	12
LH 13	+	–	6
LH 14	+	–	14
LH 15	+	–	13
LH 16	+	–	9
LH 17	+	–	11
LH 18	+	–	7
LH 19	+	–	14
LH 20	+	–	11
LH 21	+	–	13
LH 22	+	–	10
LH 23	+	–	8
LH 24	+	–	7
LH 25	+	–	12
LH 26	+	–	15

HC, healthy controls; LH-PASC; NS, nasal swabs.

**Figure 2:**
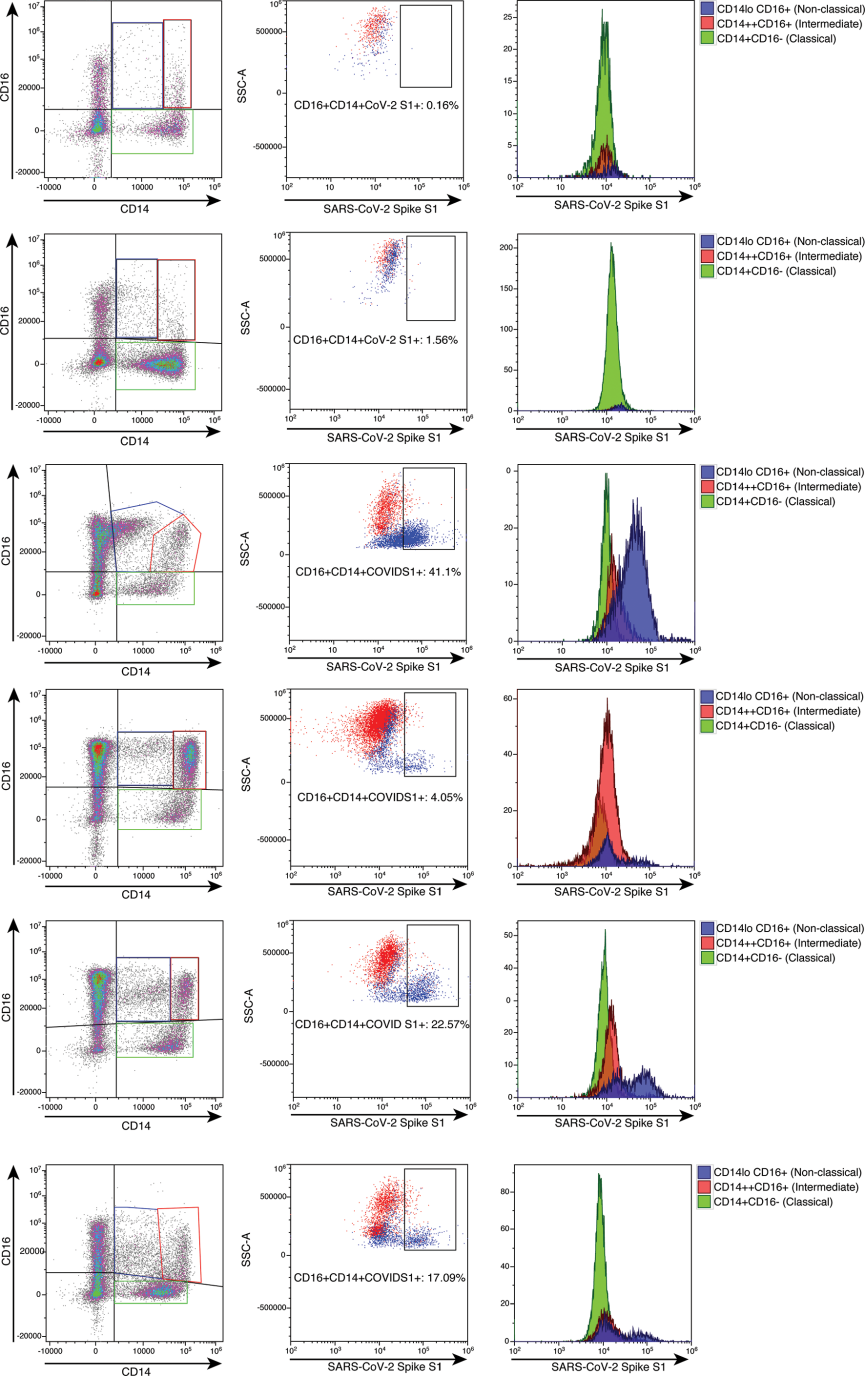
High parameter flow cytometric quantification of SARS-CoV-2 S1 protein in monocytic subsets. Cells were gated as demonstrated in **Supplementary Figure 1**. Each horizontal row represents an individual patient. A representative healthy control is demonstrated in the top row. All subsequent rows represent the spectrum of S1 containing monocytes. Classical monocytes are green, intermediate monocytes are red and non-classical monocytes are blue.

**Figure 3:**
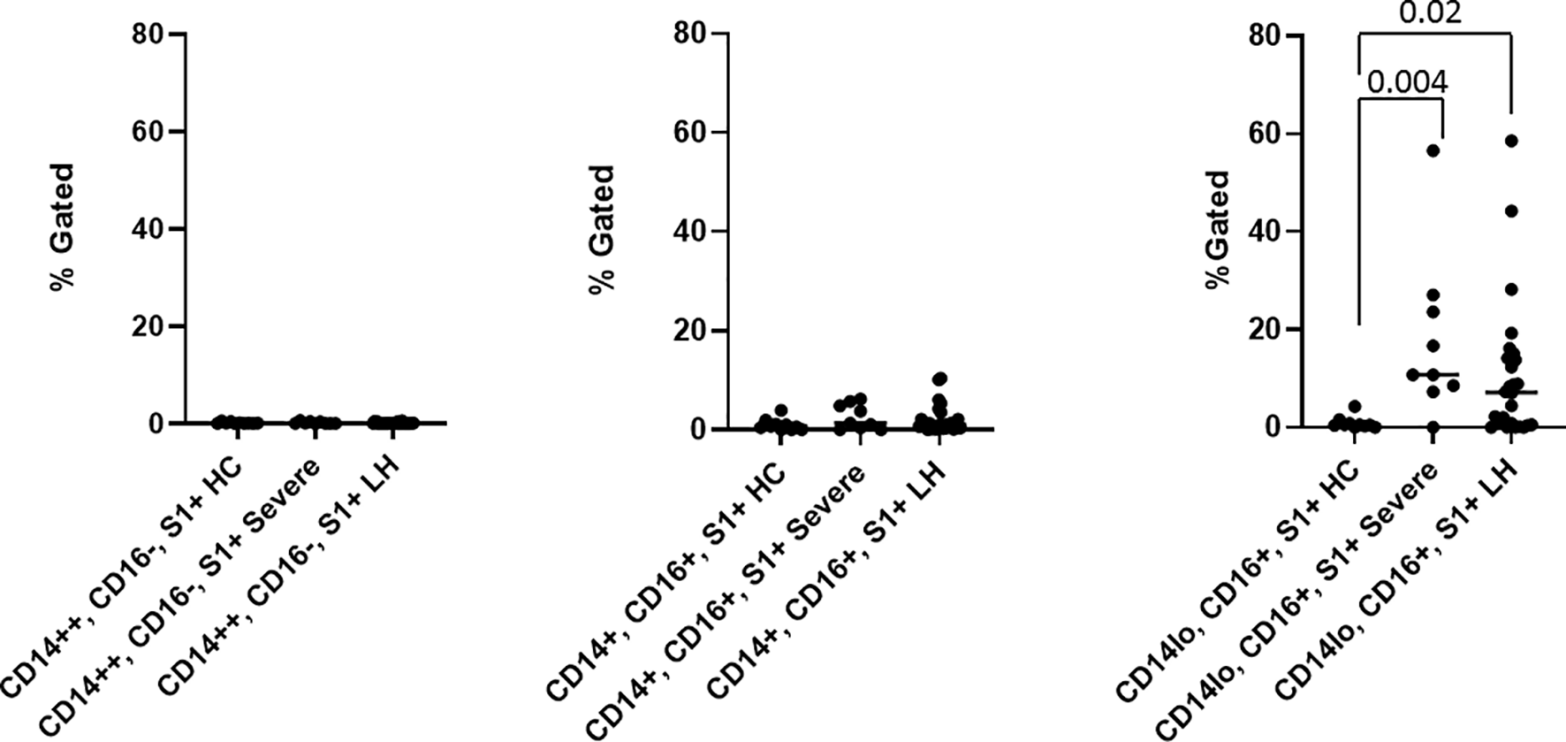
Quantification of SARS-CoV-2 S1 protein in monocyte subsets isolated from healthy 63 controls (HC), severe COVID-19 (severe), and PASC patients (LH). SARS-CoV-2 S1 protein was expressed in non-classical monocytes in both severe and PASC individuals. Values (median value-horizontal line) for each category were based on the parental gating in **Supplementary Figure 1**. Statistical analysis was performed using the Kluskal-Wallis test. P values <0.05 are considered significant.

To further establish the exact reservoir contributing to the positive signal detected using ddPCR, we performed high parameter flow cytometry with antibodies that define B cell, T-cell, and monocytic subsets in addition to simultaneous staining of these cells with an antibody for the SARS-CoV-2 S1 protein. Parental gating to identify the monocyte subsets is shown in **Supplementary Figure 1**. As demonstrated in **Figure 2**, we found distinct subpopulations of SARS-CoV-2 containing cells in the CD14lo, CD16+ monocytic subset for 73% (19 out of 26) of PASC patients and 91% (10 out of 11) of severe COVID-19 patients. As demonstrated in **Figure 3**, the quantity of SARS-CoV-2 S1 containing cells were statistically significant in both the severe patients (P=0.004) and in the PASC patients (P=0.02). Neither classical monocytes nor intermediate monocytes expressed the SARS-CoV-2 S1 protein.

**Figure 4:**
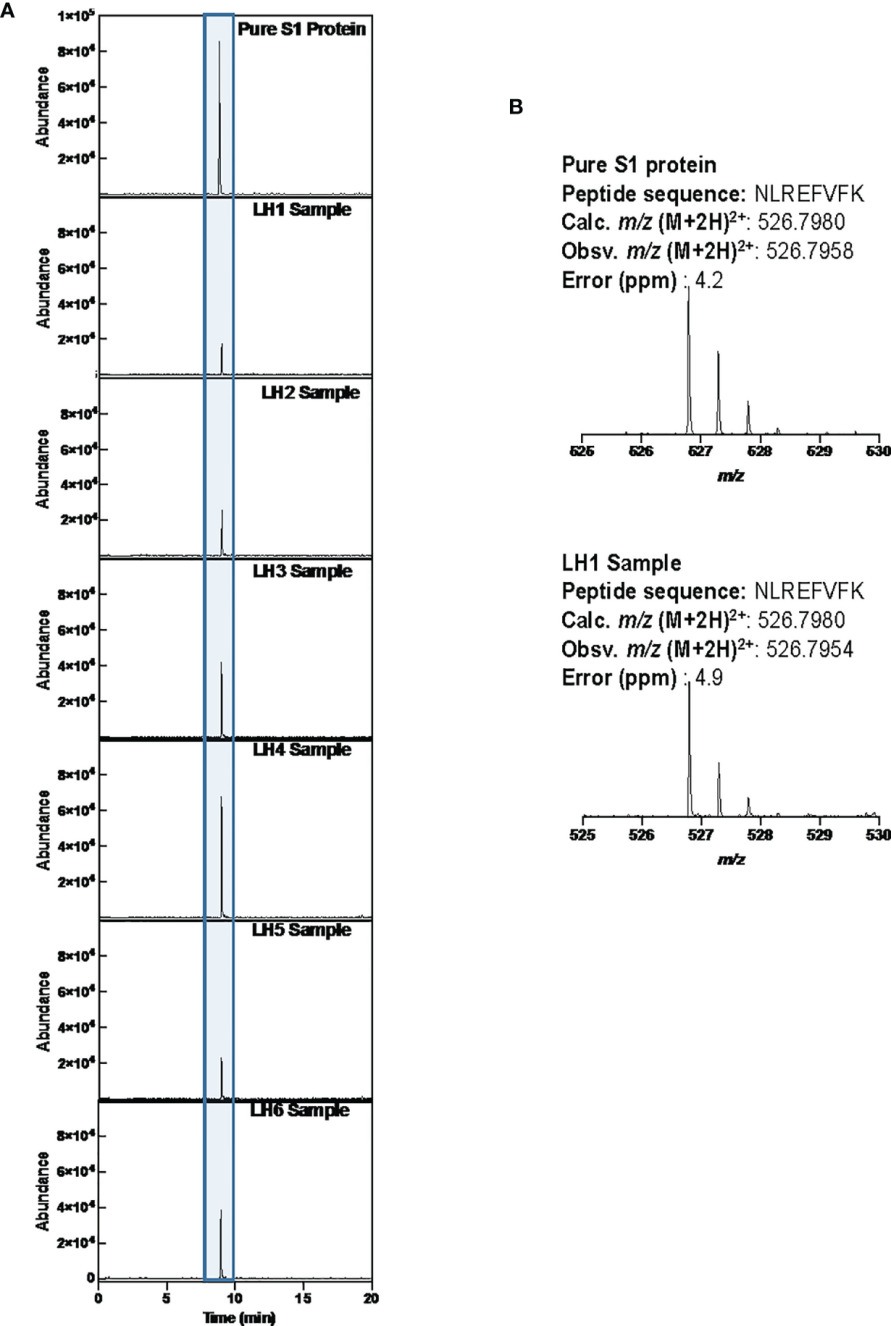
LCMS confirmation of the presence of S1 subunit in samples LH1-6. **(A)** Extracted ion chromatogram (EIC) displaying the NLREFVFK peptide. The retention time matches that of the NLREFVFK peptide in the commercial S1 standard. **(B)** Mass Spectra of the NLREFVFK from both the commercial standard and patient LH1. The Spectra show the same mass and isotope distribution.

To confirm the presence of SARS-CoV-2 S1 protein, we sorted CD14lo, CD16+ monocytes and performed Ultra High-Performance Liquid Chromatography (UHPLC). Following immunoprecipitation, the elution fractions were dried down in vacuo, resuspended in ddH_2_O and purified by to remove any non-crosslinked SARS-CoV-2 S1 antibody as well as any detergents from the commercial immunoprecipitation buffers. The digested peptides were analyzed on an Agilent 6550 IonFunnel QTOF and 1290 UHPLC by comparing patient samples to identical digests performed on commercially available SARS-CoV-2 S1 subunit. S1 subunit peptides from patient samples were mapped to a peptide database generated using commercial S1 subunit digests. Peptide identification consisted of matches in exact mass, isotope distribution, peptide charge state, and UHPLC retention time. As shown in **Figure 4**, the retention time of the representative peptide NLREFVFK in the digested commercial S1 subunit and Sample LH1-6 matched. Additionally, the Mass Spectra in **Figure 4** show identical mass, isotope distribution, and charge states for the representative peptide NLREFVFK in the representative LH1 sample and commercial S1 subunit (also observed in LH 2-6, not shown). Using these metrics, up to 44% of the S1 subunit peptides could be identified in patient samples LH1-LH6 (**Supplementary Table 1**), providing complementary evidence to flow cytometry experiments that demonstrate the presence of S1 subunit protein in these patient cells.

To determine whether the observed S1 spike protein was a product of persistent viral infection, whole viral genome sequencing was performed on monocytes from five patients of the patients who underwent LC/MS. Coverage analysis of the human control amplicons revealed adequate coverage to positively identify human genomic content. This is consistent with extraction of viral genomic content from a human host. Human controls also included targeted amplicons for amelogenin (AMELX and AMELY). The ratio of AMELX and AMELY reads is consistent with the known genders of each sample.

The sequencing coverage for the five samples was consistent with low viral titer samples or samples with high Ct values. Average coverage was between 24.17-592.87x and percent bases covered at 10x and 20x was between 10.81-19.18% and 7.69-15.24% respectively (**Table 2**). This is well below the expected threshold to eliminate stretches of Ns > 99 for consensus sequence submission to GenBank and > 90% genome coverage at 10x for accurate lineage determination and sequence submission to GISAID (www.gisaid.org). Evaluation of the reads revealed predominantly short reads (<100bp). To address poor quality reads, primer-dimers or reads that could possibly map to multiple loci, reads < MAPQ 10 were filtered resulting in the removal of 3.63-18.99% of total reads per sample.

**Table 2:** Average Coverage and Percent Bases Covered at 20x.

Sample	Average Coverage	Percent Bases Covered at 10x	Percent Bases Covered at 20x
02-03_20210625	171.64	19.18	15.24
ABA-2_20210625	59.67	14.04	10.42
BGI-2_20210625	24.17	10.81	7.69
CST-2_20210625	40.29	11.71	7.79
RG_20210625	592.87	12.6743	10.16

While the percent of bases covered varied across patients, all were less than 20% at 10X, and less at 20X coverage. In no case was full length viral genome RNA detected, consistent with a lack of replication competent viral infection.

**Figure 5:**
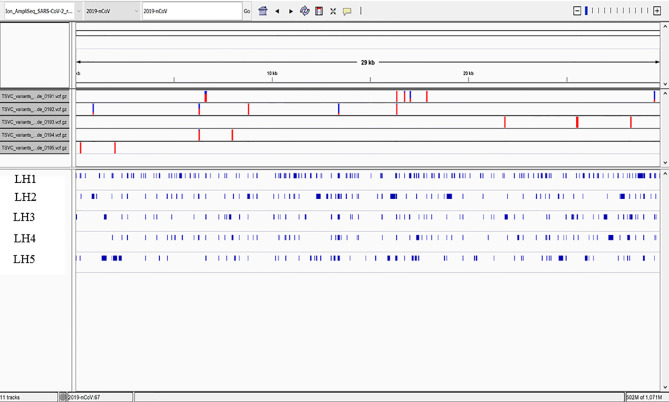
High Quality Mutations in the Callable Regions. Only fragmented viral RNA was identified in the five patients, but multiple mutations throughout the viral genome were identified, the vast majority of which were unique to each patient. Overall coverage was less than 20%, and no complete sequence in any portion of the viral genome was detected, including in the spike gene encoding the S1 subunit identified by protein analysis in these patients.

Lineage determination of the five samples from high quality mutations in the callable regions yielded lineages of B and B.1 and were non-specific due to inadequate coverage across the genome. Mutations were identified in ORF1ab in all but sample LH5. LH5 had mutations in N, S, and ORF3b (**Figure 5**).

## Discussion

Here, we report the discovery of persistent SARS-CoV-2 protein in CD14lo, CD16+ monocytes out to 15 months in some individuals and discuss the implications for the pathogenesis of PASC and severe cases of COVID-19. The three subtypes of circulating monocytes (classical, intermediate, non-classical) express very different cell surface molecules and serve very different functions in the immune system. Generally, classical’ monocytes exhibit phagocytic activity, produce higher levels of ROS and secrete proinflammatory molecules such as IL-6, IL-8, CCL2, CCL3 and CCL5. Intermediate monocytes express the highest levels of CCR5 and are characterized by their antigen presentation capabilities, as well as the secretion of TNF-α, IL-1β, IL-6, and CCL3 upon TLR stimulations. Non-classical monocytes expressing high levels of CX3CR1 are involved in complement and Fc gamma-mediated phagocytosis and anti-viral responses (6).

After maturation, human monocytes are released from bone marrow into the circulation as classical monocytes. Currently, strong evidence supports the concept that intermediate and non-classical monocytes emerge sequentially from the pool of classical monocytes (13). This is supported by transcriptome analysis showing that CD16+ monocytes have a more mature phenotype (14). In humans, 85% of the circulating monocyte pool are classical monocytes, whereas the remaining 15% consist of intermediate and non-classical monocytes (13). Classical monocytes have a circulating lifespan of approximately one day before they either migrate into tissues, die, or turn into intermediate and subsequently non-classical monocytes (6, 13).

During pathologic conditions mediated by infectious/inflammatory reactions, the proportions of monocyte subsets vary according to the functionality of each specific subpopulation (6, 13, 15). Our previous results show that during early stages of the disease, PASC group have reduced classical monocyte and increased intermediate monocyte percentages compared with healthy controls (5). We find an increase in non-classical monocytes in PASC group 6-15 months post infection, and higher percentages of intermediate and non-classical monocytes at day 0 in severe cases, suggesting augmented classical-intermediate-non-classical monocyte transition in both groups but with different kinetics.

The clinical relevance of monocyte activation in COVID-19 patients and the significance of these cells as viral protein reservoir in PASC is supported by our data reporting the presence of S1 protein within non-classical monocytes. Viral particles and/or viral proteins can enter monocyte subpopulations in distinct ways, and this appears to be regulated differently in individuals that will develop severe disease or PASC. Classical monocytes are primarily phagocytes and express high levels of the ACE-2 receptor (8). Therefore, they could either phagocyte viral particles and apoptotic virally infected cells or be potential targets for SARS-CoV-2 infection. Considering their short circulating lifespan, viral protein-containing classic monocytes turn into intermediate and non-classical monocytes. Indeed, at early stages of the disease the severe group show increased non-classical monocytes whereas in PASC both the intermediate monocytes and non-classical monocytes are elevated (5). Additionally, CD14+CD16+ monocytes express intermediate levels of ACE-2 receptors and could as well serve as an infectious target of SARS-CoV-2 as they have been shown to be an infectious target of HIV-1 and HCV^11^. Non classical monocytes have been proposed to act as custodians of vasculature by patrolling endothelial cell integrity (16). One possible explanation for S1 protein in non-classical monocytes could be that pre-existing CD14lo CD16+ cells could phagocytise virally infected apoptotic endothelial cells with subsequent degradation of the RNA and presentation of the S1 protein. Furthermore, non-classical monocytes are associated with FcR-mediated phagocytosis (17, 18), which might be related with the ingestion of opsonized viral particles after antibody production during acute infection.

Previous reports indicate that the numbers of classical monocytes decrease, but the numbers of intermediate and non-classical monocytes increase in COVID-19 patients (19). Thus, the presence of S1 protein in non-classical monocytes in both severe and PASC, might be associated with clinical characteristics and outcome of these groups. Previously, we found that individuals with severe COVID-19 have high systemic levels of IL-6, IL-10, VEGF and sCD40L (5). Consistent with our data, other studies showed association of increased production of IL-6,

VEGF and IL-10 by non-classical monocytes with disease severity (20–22).

In the case of PASC, the persistence of circulating S1-containing non-classical monocytes up to 15 months post infection, independently of the different possible mechanisms of viral proteins internalization discussed above, indicates that certain conditions may exist to maintain this cell population. It has been shown in both humans and mice that non-classical monocytes require fractalkine (CX3CL1) and TNF to inhibit apoptosis and promote their survival (22). Our previous data show high IFN-γ levels in PASC individuals (5), which can induce TNF-α production (23).

Further, TNF-α and IFN-γ induce CX3CL1/Fractalkine production by vascular endothelial cells creating the conditions that could promote survival of non-classical monocytes (24). Moreover, IFN-γ induced CX3CL1/Fractalkine production by endothelial cells (23) may create a gradient within the vascular compartment preserving non-classical monocytes expressing CX3CR1 in the circulation.

Non-classical monocytes are usually referred to as anti-inflammatory cells (22), nevertheless it was recently shown that this subset can acquire a proinflammatory phenotype (24). Non-classical monocytes have been previously shown to acquire hallmarks of cellular senescence, which promote long term survival of these cells in circulation. Additionally, this induces an inflammatory state of the non-classical monocytes that could be a manifestation of the senescence-associated secretory phenotype (SASP), characterized by a high basal NF-κB activity and production of pro-inflammatory cytokines such as IL-1α, TNF-α and IL-8 (25).

The hallmark of PASC is the heterogeneity of symptoms arising in a variety of tissues and organs. The CD14lo, CD16+, S1 protein+ monocytes could be preferentially recruited into anatomic sites expressing fractalkine and contribute to vascular and tissue injury during pathological conditions in which this monocyte subset is expanded as previously demonstrated in non-classical monocytes without S1 protein. Previously, CD16+ monocytes were demonstrated to migrate into the brain of AIDS patients expressing high levels of CX3CL1 (fractalkine) and SDF-1 (26), and mediate blood-brain barrier damage and neuronal injury in HIV-associated dementia *via* their release of proinflammatory cytokines and neurotoxic factors. Interestingly, a number of papers have been written discussing the increased mobilization of CD14lo, CD16+ monocytes with exercise (27). These data could help to explain reports of worsening PASC symptoms in individuals resuming pre-COVID exercise regimens.

In summary, the mechanism of PASC proposed in this report suggests that intermediate monocytes remain in circulation due to low CCL4 levels extending their time to differentiate leading to an accumulation of non-classical monocytes. Further, our data suggests that interruption of the CX3CR1/fractalkine pathway could be a potential therapeutic target to reduce the survival of S1-containing non-classical monocytes and the associated vascular inflammation previously discussed (5).

It is important to note that the S1 protein detected in these patients appears to be retained from prior infection or phagocytosis of infected cells undergoing apoptosis and is not the result of persistent viral replication. Full length sequencing of the five cases submitted for genomic analysis failed to identify any full-length sequence in the spike protein gene, or any other gene, that could account for the observed spike protein detected by proteomic analysis. In contrast, fragmented SARS-CoV-2 sequence was identified in all five of the cases. We have observed a pattern of high Ct value or negativity by PCR, accompanied by scant, fragmented viral sequence identified by whole viral genome sequencing over the past several months, a major shift from the low Ct value, full length viral sequences identified throughout most of 2020. The reasons for this shift are unclear, but as seen in these cases, it is unlikely these patients are producing any replication competent viral genomes, and are thus unlikely to transmit the infection. In contrast, the data reported here supports the hypothesis that an immune response to persistent viral antigens, specifically the S1 fragment of the spike protein eliciting an the PASC immune response previously published (5) and marked by elevated inflammatory markers including IFN-γ, IL-6, IL-10, and IL-2, among others.

## Data Availability Statement

The original contributions presented in the study are included in the article/**Supplementary Material**. Further inquiries can be directed to the corresponding author.

## Ethics Statement

All patients signed informed consent and this study was approved by the ethical committee of the Chronic COVID Treatment Center. The patients/participants provided their written informed consent to participate in this study.

## Author Contributions

Author contributions: RY and PP organized the clinical study and actively recruited patients. BP, AP, HR, EL, TT, PS, SH, and DM performed experiments and analyzed the data. JG-C, RM-R, JM, and XC performed the statistics and bioinformatics BP, JM, EF, JG-C, and RM-R wrote the draft of the manuscript and all authors contributed to revising the manuscript prior to submission.

## Correction note

A correction has been made to this article. Details can be found at: 10.3389/fimmu.2026.1832958.

## Conflict of Interest

BP, AP, HR, EL, and EF are employees of IncellDx, Inc. TT, PS, SH, and DM are employees of Avrok Laboratories, Inc.

The remaining authors declare that the research was conducted in the absence of any commercial or financial relationships that could be construed as a potential conflict of interest.

## Publisher’s Note

All claims expressed in this article are solely those of the authors and do not necessarily represent those of their affiliated organizations, or those of the publisher, the editors and the reviewers. Any product that may be evaluated in this article, or claim that may be made by its manufacturer, is not guaranteed or endorsed by the publisher.

## Supplementary Material

Supplementary Figure 1 Parental gating strategy used in all flow cytometric analyses. Debris was minimized using light scatter gating followed by single cell analysis to eliminate doublets. CD45 positive selection and CD19 (B-cell)/CD3 (T-cell) negative selection was also utilized to optimize monocyte selection for analysis and sorting.
