# Correction: Persistence of SARS CoV-2 S1 protein in CD16+ monocytes in post-acute sequelae of COVID-19 (PASC) up to 15 months post-infection

**DOI:** 10.3389/fimmu.2026.1832958

**Published:** 2026-04-23

**Authors:** Bruce K. Patterson, Edgar B. Francisco, Ram Yogendra, Emily Long, Amruta Pise, Hallison Rodrigues, Eric Hall, Monica Herrera, Purvi Parikh, Jose Guevara-Coto, Timothy J. Triche, Paul Scott, Saboor Hekmati, Dennis Maglinte, Xaiolan Chang, Rodrigo A. Mora-Rodríguez, Javier Mora

**Affiliations:** 1 Department of Research and Development, IncellDx Inc, San Carlos, CA, United States; 2 Department of Anesthesia, Lawrence General Hospital, Lawrence, MA, United States; 3 Department of Molecular Diagnostics, Bio-Rad Laboratories, Hercules, CA, United States; 4 Department of Allergy and Immunology, New York University (NYU) Langone Health, New York, NY, United States; 5 Lab of Tumor Chemosensitivity, Research Center on Tropical Diseases (CIET)/Research Center on Surgery and Cancer (DC) Lab, Faculty of Microbiology, Universidad de Costa Rica, San Jose, Costa Rica; 6 Department of Computer Science and Informatics (ECCI), Universidad de Costa Rica, San Jose, Costa Rica; 7 Department of Molecular Biology, Avrok Laboratories, Inc., Azusa, CA, United States; 8 Vaccine & Gene Therapy Institute and Oregon National Primate Research Center, Oregon Health & Science University, Portland, OR, United States

**Keywords:** COVID-19, PASC, SARS CoV-2 S1 protein, non-classical monocytes, CCR5, fractalkine

In the published article, there was an error. In section **Materials and Methods**, *Patients*, it was indicated that a total of 144 individuals were enrolled in the study, consisting of 29 normal individuals, 26 mild-moderate COVID-19 patients, 25 severe COVID-19 patients and 64 chronic COVID (long hauler-LH) individuals. The correct number of enrolled patients in this study is 46, consisting of: 8 healthy controls, 11 severe COVID, 1 asymptomatic, 26 post-COVID individuals.

A correction has been made to the section **Materials and Methods**, *Patients*, Paragraph 1. This sentence previously stated:

“A total of 144 individuals were enrolled in the study consisting of 29 normal individuals, 26 mild-moderate COVID-19 patients, 25 severe COVID-19 patients and 64 chronic COVID (long hauler-LH) individuals”.

The corrected sentence appears below:

“A total of 46 individuals were enrolled in the study consisting of 8 healthy controls, 11 severe COVID, 1 asymptomatic, 26 post-COVID individuals”.

The original version of this article has been updated.

