# Supplementary figures and images for: Persistence of SARS CoV-2 S1 Protein in CD16+ Monocytes in Post-Acute Sequelae of COVID-19 (PASC) up to 15 Months Post-Infection
Source: Front Immunol. 2022 Jan 10;12:746021. doi: 10.3389/fimmu.2021.746021 (PMC8784688; doi:10.3389/fimmu.2021.746021)

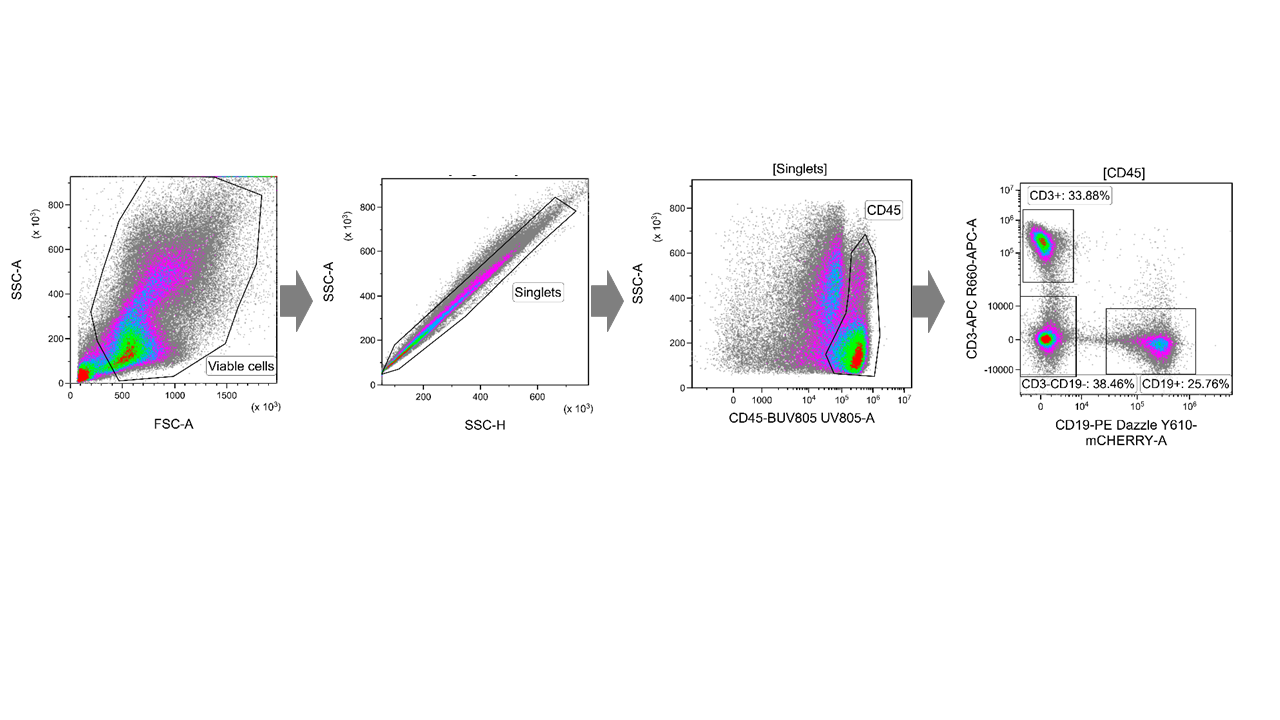

Supplement: Supplementary Figure 1 — Parental gating strategy used in all flow cytometric analyses. Debris was minimized using light scatter gating followed by single cell analysis to eliminate doublets. CD45 positive selection and CD19 (B-cell)/CD3 (T-cell) negative selection was also utilized to optimize monocyte selection for analysis and sorting. [file Image1.tif]
