# Supplementary material for: Persistence of SARS CoV-2 S1 Protein in CD16+ Monocytes in Post-Acute Sequelae of COVID-19 (PASC) up to 15 Months Post-Infection
Source: Front Immunol. 2022 Jan 10;12:746021. doi: 10.3389/fimmu.2021.746021 (PMC8784688; doi:10.3389/fimmu.2021.746021)
Supplement: Supplementary file 2 [file DataSheet1.pdf]

No.	Position	Sequences
1	27-37	VFRSSVLHSTQ
2	32-37	VLHSTQ
3	65-72	DNPVLPFN
4	63-82	RFDNPVLPFNDGVYFASTEK
5	70-82	PFNDGVYFASTEK
6	73-82	DGVYFASTEK
7	96-98	DSK
8	163-167	DLEGK
9	168-172	QGNFK
10	173-180	NLREFVFK
11	174-180	LREFVFK
12	181-182	NI
13	183-187	DGYFK
14	192-199	HTPINLVR
15	213-237	DLPIGINITRFQTLLALHRS YLTPG
16	272-274	DAV
17	275-278	DCAL
18	272-285	DAVDCALDCALDPLSETK
19	281-289	LSETKCTLK
20	290-295	SFTVEK
21	342-348	RISNCVA
22	364-371	CYGVSPTK
23	374-386	DLCFTNVYADSFV
24	372-388	LNDLCFTNVYADSFVIRGDEVRQIAPG
25	383-389	DSFVIRG
26	403-404	IA
27	405-409	DYNYK
28	403-409	IADYNYK
29	405-411	DYNYKLP
30	413-426	DFTGCVIAWNSNNL
31	427-429	DSK
32	430-439	VGGNYNYLYR
33	448-451	PFER
34	514-520	KSTNLVK
35	515-520	STNLVK
36	523-542	CVNFNFNGLTGTGVLTESNK
37	523-543	CVNFNFNGLTGTGVLTESNKK
38	544-552	FLPFQQFGR
39	544-555	FLPFQQFGRDIA
40	546-552	PFQQFGR
41	546-555	PFQQFGRDIA
42	559-562	DAVR
43	563-570	DPQTLEIL
44	612-619	DQLTPTWR

45	612-657	DQLTPTWRVYSTGSNVFQTR AGCLIGAEHVNNSYECDIPIGAGICA
46	648-657	DIPIGAGICA

Detected from the purified protein			Detected from Patient		Peak Intensity
Mass	$[M+H]^+$	Rotention Time	$[M/z+H]^+$	Charge	Sample 1
1259.6695	1260.677	11.46	630.8222	2+	1.808E+05
683.3595	684.3673	6.15	342.6699	2+	1.745E+04
914.457	915.4648	18.47	458.2172	2+	1.278E+04
2315.1237	2316.132	23.97	772.7145	3+	x
1473.6768	1474.685	17.38	737.8457	2+	x
1115.5207	1116.529	12.35	558.7509	2+	x
348.1718	349.1796	11.48	175.0872	2+	5.290E+04
560.2878	561.2956	5.5	561.2878	1+	1.997E+04
592.3042	593.312	7.88	297.1422	2+	3.058E+04
1051.5887	1052.597	8.96	526.7962	2+	5.717E+04
937.54	938.5478	22.23	469.7794	2+	2.649E+04
245.1448	246.1526	1.14	246.145	1+	2.526E+04
628.2929	629.3007	19.17	315.1534	2+	9.022E+03
948.5577	949.5655	14.8	475.2864	2+	1.656E+04
2808.5722	2809.58	24.13	937.1902	3+	x
303.1503	304.1581	4.98	304.1509	1+	1.537E+06
477.1966	478.2044	12.35	478.1886	1+	x
1532.7036	1533.711	16.33	767.3572	2+	x
1021.53	1022.538	11.48	511.7731	2+	1.901E+07
709.3719	710.3797	9.5	710.364	1+	2.695E+03
818.4141	819.4219	12.39	410.2057	2+	5.351E+04
910.4291	911.4369	9.69	456.2201	2+	1.222E+06
1492.6672	1493.675	13.03	747.3352	2+	1.523E+03
2045.987	2046.995	31.26	1023.9987	2+	4.717E+03
792.4202	793.428	11.18	397.2144	2+	1.015E+05
202.139	203.1468	4.7	203.1408	1+	1.598E+04
701.3093	702.3171	9.31	351.6528	2+	8.901E+04
885.424	886.4318	11.04	443.7209	2+	1.626E+04
911.436	912.4438	14.92	456.7217	2+	6.918E+04
1609.7267	1610.735	25.38	805.877	2+	1.002E+04
348.1718	349.1796	11.44	349.1841	1+	6.750E+06
1217.5792	1218.587	12.84	609.7855	2+	7.934E+03
547.2827	548.2905	7.28	548.274	1+	5.134E+03
788.474	789.4818	10.5	395.2259	2+	4.663E+03
660.3879	661.3957	4.93	331.1923	2+	6.319E+04
2171.039	2172.047	17.93	724.6711	3+	3.216E+04
2299.1267	2300.135	24.6	767.3803	3+	x
1138.5996	1139.607	14.72	570.2873	2+	3.151E+04
1437.7435	1438.751	16.52	719.8711	2+	9.352E+03
878.4396	879.4474	18.5	440.2128	2+	1.575E+04
1177.5887	1178.597	22.71	589.7847	2+	6.565E+03
459.2514	460.2592	5.8	460.2429	1+	2.088E+05
927.4986	928.5064	21.42	928.498	1+	3.482E+03
1015.5088	1016.517	15.3	508.7594	2+	1.107E+04

4926.2802	4927.288	22.02	1232.5713	4+	9.719E+03
985.483	986.4908	21.22	493.7432	2+	1.982E+04

Sample 2	Sample 3	Sample 4	Sample 5	Sample 6
1.769E+05	2.203E+05	1.656E+05	4.874E+04	2.169E+05
1.589E+04	1.350E+04	1.784E+04	2.152E+04	1.889E+04
2.158E+04	3.455E+04	2.094E+04	2.738E+04	2.220E+04
4.101E+03	1.217E+04	8.495E+03	9.346E+03	x
x	1.617E+04	4.575E+03	1.183E+04	x
4.067E+03	5.732E+03	2.266E+04	1.409E+04	6.881E+03
6.324E+04	7.220E+04	5.870E+04	1.639E+05	8.167E+04
2.197E+04	2.551E+04	1.332E+05	1.084E+05	1.631E+04
1.723E+04	3.286E+04	3.380E+04	1.705E+04	3.796E+04
8.879E+04	1.554E+05	2.275E+05	8.242E+04	1.476E+05
2.985E+04	3.496E+04	6.535E+04	5.821E+04	4.626E+04
6.399E+03	1.988E+04	9.223E+03	4.179E+04	2.286E+04
1.040E+04	1.347E+04	1.329E+04	2.176E+04	1.139E+04
1.721E+04	1.008E+04	2.537E+04	2.122E+04	2.065E+04
x	5.500E+03	8.609E+03	1.423E+04	4.446E+03
1.407E+06	1.464E+06	1.418E+06	1.295E+06	4.981E+05
x	4.533E+04	1.090E+04	6.507E+03	x
x	6.123E+03	7.722E+03	1.660E+04	x
1.672E+07	1.987E+07	1.573E+07	6.078E+06	1.817E+07
2.467E+03	2.373E+03	1.731E+03	x	1.978E+03
4.052E+04	8.367E+05	1.616E+05	1.818E+05	2.209E+04
1.304E+06	1.252E+06	1.248E+06	1.406E+06	1.225E+06
4.927E+03	2.662E+03	4.103E+03	5.292E+03	4.571E+03
1.011E+04	5.340E+04	2.073E+04	2.083E+04	2.559E+03
1.018E+05	1.276E+05	5.225E+05	3.503E+05	7.019E+04
1.930E+04	2.912E+04	2.263E+04	2.229E+04	1.991E+04
9.015E+04	8.309E+04	8.734E+04	9.850E+04	7.720E+04
3.843E+04	2.013E+04	4.090E+04	3.193E+04	3.519E+04
4.840E+04	5.857E+05	2.030E+05	2.381E+05	3.273E+04
1.103E+04	1.482E+04	2.652E+04	4.200E+04	1.453E+04
7.032E+06	8.125E+06	8.544E+06	8.371E+06	7.452E+06
6.525E+03	6.022E+03	1.922E+04	9.391E+03	7.370E+03
3.548E+03	7.167E+04	2.117E+04	1.858E+03	2.796E+03
9.453E+03	7.707E+03	2.175E+04	4.092E+04	9.452E+03
5.495E+04	4.786E+04	6.364E+04	3.018E+04	6.595E+04
2.198E+04	3.136E+04	4.001E+04	2.229E+04	5.302E+04
x	x	4.706E+03	1.051E+04	x
2.070E+04	1.490E+05	1.148E+05	4.230E+04	1.843E+04
5.572E+03	4.280E+04	2.321E+04	1.096E+05	4.708E+03
4.693E+03	9.308E+03	5.106E+03	1.582E+04	1.322E+04
1.047E+04	5.887E+04	2.925E+04	1.749E+04	3.925E+03
1.946E+05	1.439E+06	4.090E+05	2.855E+05	1.502E+05
x	5.044E+03	2.176E+03	x	3.033E+03
x	1.842E+04	7.772E+03	5.101E+03	1.234E+04

9.259E+03	1.295E+04	1.161E+04	6.864E+03	1.030E+04
3.112E+03	6.618E+03	3.550E+03	1.112E+04	6.703E+03
